# Exploring Masticatory and Occlusal Factors in Burning Mouth Syndrome: A Scoping Review

**DOI:** 10.3390/jcm15103633

**Published:** 2026-05-09

**Authors:** Ilenia Dorigo, Katia Rupel, Luca Pellegrini, Giulia Ottaviani, Bachar Reda

**Affiliations:** Department of Medical, Surgical and Health Sciences, University of Trieste, 34149 Trieste, Italy; ilenia.dorigo@studenti.units.it (I.D.); krupel@units.it (K.R.); luca.pellegrini@units.it (L.P.); rbachar@units.it (B.R.)

**Keywords:** Burning Mouth Syndrome, BMS, occlusal dysesthesia, occlusion, dental tactile acuity, oral sensory alterations

## Abstract

**Background/Objectives**: Burning Mouth Syndrome (BMS) is a chronic condition characterized by recurrent oral burning in the absence of visible mucosal lesions. Although its etiology is multifactorial and not fully understood, recent evidence suggests that alterations in masticatory function and occlusal patterns may contribute to symptom development and persistence. The aim of this review is to explore the association between BMS and alterations in masticatory and occlusal function and perception. **Methods**: A scoping review was conducted using PubMed, Cochrane, Web of Science, and Scopus databases. The search covered studies published between January 1988 and February 2026. Inclusion criteria included studies in English addressing the relationship between BMS and masticatory or occlusal function and perception. Search strategy included both primary and review studies, and excluded congress abstracts and proceedings, commentaries, letters, theses/dissertations, editorials, animal and preclinical studies. **Results**: Thirteen studies met the inclusion criteria and were grouped into three categories: occlusal perception and sensory alterations (*n* = 4), dysfunctional oral habits (*n* = 6), and prosthetic factors (*n* = 3). Findings, presented descriptively, suggest that BMS patients may exhibit altered occlusal and sensory perception, increased sensitivity to mechanical or thermal stimuli, and possible central pain dysregulation. Dysfunctional or parafunctional habits may also be associated with symptom persistence. Additionally, prosthetic factors were reported in some studies to be linked with oral discomfort, with symptom improvement following corrective interventions in selected cases. **Conclusions**: BMS appears to be a complex and multifactorial condition. Altered sensory perception, parafunctional habits, and prosthetic factors may contribute to symptom expression. However, the available evidence is heterogeneous and limited; therefore, further well-designed longitudinal and interventional studies are needed before definitive conclusions can be drawn.

## 1. Introduction

Burning Mouth Syndrome (BMS) is a chronic, idiopathic orofacial pain condition, classified as an intraoral burning or dysesthetic sensation occurring daily for more than two hours per day for over three months, without identifiable causative lesions on clinical examination or investigation [1,2]. Its prevalence is estimated between 1% and 5%, with a marked predominance in postmenopausal women, highlighting its clinical and social relevance [3,4].

The etiology of BMS is multifactorial, with neuropathic, psychological, endocrine, and local factors proposed as potential determinants [3,5]. However, it is essential to clearly distinguish between primary BMS, which is an idiopathic diagnosis of exclusion, and secondary burning symptoms, which are attributable to identifiable local or systemic conditions. This distinction is critical, as a number of local, prosthetic, or behavioral factors—such as oral parafunctions, denture-related issues, or mucosal irritation—may mimic, exacerbate, or coexist with burning symptoms without representing primary BMS. Failure to maintain this conceptual separation may lead to diagnostic ambiguity in a condition whose definition relies heavily on the exclusion of identifiable causes [2].

Although the hallmark symptom is a burning sensation, the clinical presentation is often heterogeneous. Patients may report oral tingling, dysgeusia, xerostomia, or nonspecific intraoral discomfort. In some cases, symptoms may manifest as masticatory discomfort or orofacial pain resembling temporomandibular disorders (TMD) or myofascial pain, potentially leading to misdiagnosis and inappropriate or even harmful treatments if secondary causes are not adequately ruled out [4,5].

An expanded diagnostic framework has been proposed to capture patients presenting with dysaesthetic (e.g., tingling, taste disturbance, xerostomia) and perceptual symptoms (e.g., foreign body sensation, altered tongue perception) without burning, yet sharing key features with BMS. This subgroup has been termed Oral Dysaesthetic and Perceptual Disorder (ODPD), emphasizing the need to broaden current diagnostic criteria [6].

Diagnosis remains largely clinical and based on exclusion, requiring careful evaluation to rule out local mucosal disease, nutritional deficiencies, salivary gland dysfunction, metabolic or hormonal disorders, and neuropathies [7]. Quantitative sensory testing and neurophysiological assessments have revealed alterations in somatosensory processing consistent with a neuropathic basis for BMS [5,8].

Although recent advances have contributed to a better understanding of the condition and several therapeutic strategies have been explored, including topical/systemic clonazepam, alpha-lipoic acid, capsaicin, low-level laser therapy, and cognitive-behavioral therapy [9,10]. A significant proportion of patients exhibit only partial responses or poor tolerance to systemic medications, highlighting the need for individualized and multimodal management strategies [9].

Several mechanisms, including altered sensory processing, parafunctional habits, and prosthetic factors, have been proposed to contribute to burning symptomatology. However, these factors should be interpreted within the appropriate diagnostic framework, distinguishing between causal conditions and modifiers of symptom expression. Current evidence remains fragmented and has not yet been comprehensively synthesized, emphasizing the need for further investigation.

The objective of this scoping review is to explore the possible correlations between BMS onset and/or changes in burning symptoms and occlusion issues including occlusal sensory alterations, occlusal dysfunctional habits and prosthetic issues.

## 2. Materials and Methods

This scoping review was conducted following the Joanna Briggs Institute (JBI) methodology to map and synthesize the available evidence [10]. Reporting adhered to the PRISMA extension for Scoping Reviews (PRISMA-ScR) [11]. The protocol was not registered.

### 2.1. Population–Concept–Context Framework

The review was guided by the Population–Concept–Context (PCC) framework:

- Population: patients diagnosed with Burning Mouth Syndrome (BMS)
- Concept: masticatory function, occlusion, parafunctional habits, and prosthetic factors
- Context: clinical observational, interventional and review studies

### 2.2. Search Strategy

A comprehensive literature search was conducted in MEDLINE (via PubMed), Cochrane Library, Web of Science, and Scopus up to February 2026. Both controlled vocabulary (MeSH terms) and free-text keywords were used.

The search combined terms related to:

- Population: “Burning Mouth Syndrome” OR “BMS”
- Concepts: mastication, dental occlusion, parafunctional habits, and prosthetic factors

Search strategies adapted for each database are provided in Supplementary Table S1.

### 2.3. Article Screening and Data Extraction

All retrieved records were exported and duplicates were removed. Study selection was performed in two stages: title/abstract screening followed by full-text assessment.

Eligibility criteria were applied consistently throughout the selection process. Studies were included if they were original studies (observational, interventional or systematic review studies) published in English between January 1988 and February 2026. Exclusion criteria were narrative reviews, conference abstracts, editorials, letters, theses, animal or preclinical studies, and studies with non-extractable data.

**Figure 1:**
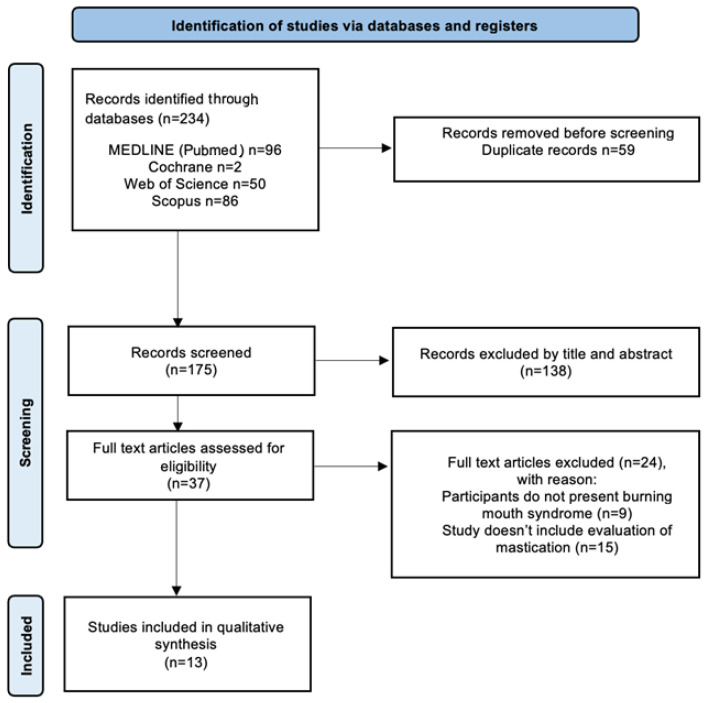
Systematic review flowchart following the PRISMA-ScR (Preferred Reporting Items for Systematic Reviews and Meta-Analyses) reporting guidelines.

Screening was conducted independently by two reviewers (KR, ID) following calibration exercises (target agreement ≥ 90%). Discrepancies were resolved by discussion or by consulting a third reviewer (BR). Reasons for exclusion at the full-text stage were recorded. The selection process is illustrated in the PRISMA flow diagram (Figure 1).

Data extraction was performed using a standardized form and included: study characteristics (authors, year, design), population details, exposures/interventions, outcomes, and main findings.

### 2.4. Critical Appraisal

Consistent with scoping review methodology, no formal risk-of-bias assessment was conducted, as the objective was to map the available evidence rather than to evaluate study quality.

## 3. Results

### 3.1. Study Selection

The database search identified a total of 234 records. The number of records retrieved from each database was: PubMed (*n* = 96), Cochrane Library (*n* = 2), Scopus (*n* = 86), Web of Science (*n* = 50). After removal of duplicates, 175 records were screened for eligibility by title and abstract, and 138 were excluded for irrelevance. A total of 37 full-text articles were assessed for eligibility. Of these, 24 studies were excluded after full-text evaluation. The detailed list of excluded studies with reasons for exclusion is provided in Supplementary Table S2. Ultimately, 13 articles were included in the review.

The selection process is summarized in the PRISMA-ScR flow diagram (Figure 1).

### 3.2. Study Characteristics

The included studies comprised a heterogeneous body of evidence, including four case–control studies, one observational study, one clinical study, two retrospective studies, one prospective study, three literature reviews, and one hypothesis paper.

Given this methodological heterogeneity, findings were synthesized descriptively, and no hierarchical weighting of evidence was applied.

### 3.3. Thematic Synthesis: Distinguishing Clinical and Theoretical Evidence

The 13 included studies were analyzed by distinguishing between contributions from direct clinical research (case–control, observational, prospective, and retrospective studies) and those derived from theoretical synthesis (literature reviews and hypothesis papers).

#### 3.3.1. Occlusal Perception and Sensory Alterations

In this category, all included studies (*n* = 4) [12,13,14,15] are clinical and experimental in nature:

- Case–Control Data: Research conducted on patients identified objective alterations in tactile and thermal thresholds, suggesting central sensitization and the involvement of small nerve fibers [12,13].
- Tactile Acuity: One case–control study demonstrated that BMS patients exhibit increased occlusal tactile acuity, perceiving extremely thin contacts (8–48 µm), indicating potential trigeminal pathway involvement [14].
- Functional Response: Clinical testing showed that mechanical stimulation through gum chewing can influence plasma adrenaline levels and temporarily reduce pain scores [15].

Details of these studies are summarized in Table 1.

#### 3.3.2. Dysfunctional Oral Habits

For this theme (*n* = 6) [16,17,18,19,20,21], findings emerge from a combination of field observations and conceptual frameworks:

- Contributions from Reviews and Hypothesis Papers [16,17,18]: Half of the studies in this section (*n* = 3) are reviews or theoretical models. They suggest that chronic stress and altered oral motor awareness sustain symptoms, though they do not provide new direct experimental measurements within these specific papers
- Evidence from Clinical Studies (Observational/Retrospective) [19,20,21]: Data collected directly from patient cohorts show a strong correlation between BMS symptom severity and the presence of parafunctions such as clenching, grinding, and tongue pressing.

Details are provided in Table 2.

#### 3.3.3. Prosthetic-Related Factors

The evidence in this area (*n* = 3) [22,23,24] is exclusively clinical-observational:

- Prospective and Retrospective Data [22,23,24]: Studies documented significant pain reduction following the correction of inadequate dentures or the re-establishment of occlusal balance.
- Hypersensitivity [22]: Clinical investigations identified a link between oral burning and contact hypersensitivity to specific dental materials in selected patient subsets.

Details are summarized in Table 3.

To synthesize the findings across the three thematic categories, Table 4 summarizes the main concepts, themes, and mapped categories identified in the included studies. It highlights the conceptual focus of each research area, the predominant findings, the methodological diversity, and the principal knowledge gaps.

### 3.4. Critical Synthesis of Evidence

The mapping of the literature reveals a clear distinction between empirical data and conceptual frameworks. While the roles of sensory alterations and prosthetic factors are supported by original clinical data, such as measured tactile thresholds and documented recovery after clinical intervention, the evidence regarding dysfunctional oral habits is more balanced between clinical observations and theoretical or review-based papers.

Key observations from this critical appraisal include:

- Methodological Heterogeneity: The evidence for sensory alterations (Theme 1) and prosthetic issues (Theme 3) relies primarily on clinical and experimental designs.
- Theoretical Prevalence: In contrast, the link between parafunctional habits and BMS (Theme 2) is heavily supported by literature reviews and hypothesis papers, which propose multifactorial models but often lack new longitudinal experimental data.
- Nature of Findings: Most clinical findings across all themes remain hypothesis-generating rather than definitive due to small sample sizes and the predominance of cross-sectional designs.

## 4. Discussion

This scoping review aimed to map the current evidence on the relationship between BMS and alterations in masticatory and occlusal function and perception, identifying recurring findings, conceptual gaps, potential directions for future research and clinical implications.

Thirteen studies were included (four case–control, one clinical, one observational, two retrospective, one prospective, three reviews, one hypothesis paper). The evidence was organized into three themes:

(1) occlusal perception and sensory alterations in BMS,

(2) oral dysfunctional habits and BMS,

(3) prosthetic-related factors and BMS.

(1) Occlusal perception and sensory alterations in BMS

Several studies have explored the clinical and neurophysiological characteristics of BMS, particularly in relation to the stomatognathic system. Case–control studies by De Siqueira et al. (2013, 2014) and Canfora et al. (2024), together with an experimental study by Sekine et al. (2020), reported altered pain thresholds and tactile discrimination, suggesting possible involvement of small nerve fibers and central sensitization [12,13,14,15]. De Siqueira et al. observed generalized somatosensory abnormalities [12,13], while Canfora et al. reported increased occlusal tactile acuity, particularly for thin contacts (8–48 µm) [14], suggesting possible involvement of trigeminal pathways. Sekine et al. demonstrated that masticatory stimulation through gum chewing may reduce pain scores and influence plasma adrenaline levels, indicating a potential interaction between functional activity and neuroendocrine responses [15]. However, these findings are based on a small sample and heterogeneous methodologies, and a lack of longitudinal confirmation. Therefore, they should be considered hypothesis-generating rather than conclusive. Overall, the available evidence may indicate the presence of both generalized and localized sensory alterations in BMS, although causal relationships cannot be established.

(2) Oral dysfunctional habits and BMS

Several clinical studies have reported an increased prevalence of parafunctional habits and signs of temporomandibular disorders among patients with BMS [20]. Associations between BMS, psychological distress, and oral behaviors have also been observed in observational studies [21]. However, these findings are largely derived from cross-sectional designs, limiting the ability to establish temporal or causal relationships.

Theoretical and narrative contributions have proposed mechanisms linking parafunctional behaviors—such as clenching, grinding, and tongue pressing—to altered neuromuscular balance and pain perception [16,19]. In addition, some authors have suggested that emotional stress may contribute to compulsive masticatory activity [17] and that altered mastication could modulate pain perception in chronic orofacial conditions [18].

Taken together, the evidence remains heterogeneous and is characterized by a predominance of cross-sectional studies and conceptual frameworks. As such, it is not possible to determine whether parafunctional habits represent predisposing factors, consequences of BMS, or coexisting behaviors. The absence of standardized assessment methods and the lack of longitudinal or interventional studies further limit the strength of current conclusions.

(3) Prosthetic-related factors and BMS

Local factors, including dental prostheses and hypersensitivity to dental materials, have also been investigated as possible contributors to BMS symptoms. Some studies reported associations between poorly adapted prostheses and oral discomfort, as well as symptom improvement following prosthetic adjustment. Differences in prosthetic function, occlusal balance, and hypersensitivity to dental materials have also been described [22,23,24].

These findings suggest that local mechanical or material-related factors may play a role in symptom expression in a subset of patients. However, most studies focused on isolated factors, did not account for neurological or psychological variables, and lacked long-term follow-up. Therefore, these factors cannot be considered causal and should be interpreted cautiously within a multifactorial framework.

Across the included studies, BMS patients were frequently reported to present altered tactile and thermal sensitivity, changes in occlusal perception, and features commonly described as neuropathic pain. Parafunctional behaviors were commonly and often associated with psychological distress and temporomandibular disorders. In addition, prosthetic factors and contact hypersensitivity were potential local aggravating factors. However, most studies focused on isolated factors, did not account for neurological or psychological variables, and lacked long-term follow-up. Therefore, these factors cannot be considered causal and should be interpreted cautiously within a multifactorial framework.

The findings of this review should be considered in light of its strengths and limitations. A major strength lies in its integrative and multidisciplinary perspective, combining neurophysiological, psychological, and dental findings across different study designs. The thematic organization allowed a structured overview of the literature and facilitated the identification of key research areas.

However, the available evidence remains limited due to small sample sizes, heterogeneous methodologies, and the absence of standardized diagnostic protocols. The variability in study design and outcomes prevented quantitative synthesis, and differences in inclusion criteria may have introduced selection bias. Furthermore, the predominance of cross-sectional and short-term studies limits the ability to assess causality and long-term outcomes.

From a clinical perspective, some authors suggest that the evaluation of BMS may benefit from a multidisciplinary approach, including dental, neurological, and psychological assessment. However, the current evidence base is insufficient to support specific management recommendations, and any clinical implications should be considered exploratory.

Future research should aim to adopt standardized diagnostic criteria and longitudinal or interventional study designs to better clarify the relationship between BMS, sensory alterations, and functional factors. The investigation of neurophysiological and biochemical parameters, including potential biomarkers, may provide further insights, although current evidence is insufficient to support their routine clinical use. A multidisciplinary research approach integrating dental, neurological, and psychological data may help to better understand the complexity of BMS.

In conclusion, the current evidence suggests that BMS may involve multiple interacting factors, including sensory, behavioral, and prosthetic components. However, the available data remain limited and heterogeneous, and the findings of this review should be considered hypothesis-generating rather than definitive.

## 5. Conclusions

The available evidence suggests that BMS may be associated with a range of interacting factors, including sensory alterations, psychological components, and functional or mechanical disturbances within the stomatognathic system. However, the current literature is limited by small sample sizes, heterogeneous methodologies, and predominantly cross-sectional designs, which prevent causal inferences. As a result, the findings of this scoping review should be considered exploratory and hypothesis-generating rather than definitive.

Future research may benefit from longitudinal and controlled interventional designs and the development of standardized diagnostic and functional assessment protocols. The exploration of neurochemical and sensory biomarkers (such as adrenaline or serotonin) may represent a promising area for further investigation, but current evidence is insufficient to support their routine use. Ultimately, adopting a multidisciplinary research approach that integrates dental, neurological, and psychological perspectives may help to better understand the complexity of BMS, although further high-quality studies are needed before clinical recommendations can be drawn.

## Figures and Tables

**Table 1:** Studies reporting occlusal perception and sensory alterations in BMS patients.

**Authors (Year)**	De Siqueira et al. (2013) [12]	De Siqueira et al. (2014) [13]	Canfora et al. (2024) [14]	Sekine (2020) [15]
**Study Design**	Case–control	Case–control	Case–control	Clinical study
**Population**	BMS patients vs. healthy controls	BMS patients vs. healthy controls	BMS patients vs. healthy controls	30 BMS patients
**Intervention/Focus**	Evaluation of tactile and thermal sensitivity, occlusal perception	Neurochemical modulation of orofacial pain	Analysis of masticatory function and occlusal balance	Assessment of sensory and motor components
**Key Findings**	Altered tactile thresholds and increased thermal sensitivity; central sensitization suggested	Dysfunction in central pain modulation pathways observed	Altered occlusal contact and reduced chewing efficiency in BMS patients	Altered proprioceptive responses and irregular occlusal patterns detected

**Table 2:** Studies reporting correlations between dysfunctional oral habits and BMS.

**Authors (Year)**	Scala et al. (2003) [16]	Toyofuku et al. (2016) [17]	Zakrzewska (2013) [18]	Grushka et al. (2003) [19]	Corsalini et al. (2013) [20]	Chimenos-Küstner et al. (2017) [21]
**Study Design**	Review	Literature review	Literature review	Hypothesis paper	Observational study	Retrospective study
**Population**	BMS patients (literature-based)	BMS patients (literature-based)	BMS patients (literature-based)	Conceptual analysis	40 BMS patients	BMS cohort
**Intervention/Focus**	Overview of BMS pathophysiology and parafunctions	Neuropsychological aspects of BMS and stress-related habits	Clinical overview of BMS etiology and risk factors	Theoretical model of oral motor awareness and central mechanisms	Assessment of parafunctional behaviors (clenching, grinding)	Analysis of orofacial dysfunctions and BMS correlation
**Key Findings**	Oral parafunctions and stress are key triggers of BMS symptoms.	Chronic stress and oral motor dysfunctions sustain BMS symptoms.	BMS etiology is multifactorial with neural and behavioral contributions.	Highlighted relevance of oral motor awareness and perception.	Strong correlation between parafunctions and symptom severity.	Multifactorial link between masticatory dysfunction and BMS symptoms.

**Table 3:** Studies describing prosthetic issues in BMS patients.

**Authors (Year)**	Lamey et al. (1988) [22]	Svensson et al. (1995) [23]	Dal Sacco et al. (2005) [24]
**Study Design**	Prospective study	Case–control study	Retrospective study
**Population**	Denture wearers with burning symptoms	BMS patients vs. controls	BMS patients with prosthetic restorations
**Intervention/Focus**	Evaluation of denture fit and material sensitivity	Analysis of prosthetic parameters and occlusal balance	Assessment of outcomes after new prosthetic fabrication
**Key Findings**	Inadequate denture fit linked to burning symptoms; improvement after correction	Poor occlusal balance and excessive mucosal pressure associated with oral pain	Pain reduction after prosthetic correction; hypersensitivity to materials identified

**Table 4:** Summary of the main concepts, themes, and mapped categories identified in the included studies.

**Theme**	Sensory and Occlusal Alterations [12,13,14,15]	Dysfunctional Oral Habits [16,17,18,19,20,21]	Prosthetic Factors [22,23,24]
**Conceptual focus**	Central and peripheral sensory processing, occlusal function, and masticatory performance	Parafunctional behaviors (clenching, grinding), stress-related mechanisms, and psychogenic factors	Quality and adaptation of dental prostheses, occlusal balance, mucosal overload
**Main findings**	BMS patients show altered tactile and thermal thresholds, irregular occlusal patterns, and possible central sensitization.	Chronic oral habits and stress maintain or exacerbate BMS symptoms; multifactorial model supported.	Poorly fitting or imbalanced prostheses correlate with oral burning; symptom improvement after prosthetic correction.
**Methodological variety**	Case–control and clinical studies, small to medium samples	Reviews, observational, retrospective, and hypothesis-based studies	Prospective, case–control, and retrospective designs
**Identified gaps**	Lack of neuroimaging and longitudinal data confirming causality; limited sample diversity	Absence of standardized behavioral assessment tools; heterogeneity in diagnostic criteria	Limited standardization of prosthetic parameters; small samples; lack of controlled follow-ups

## Data Availability

Data are available upon request to the corresponding author.

## Supplementary Materials

The following supporting information can be downloaded at: https://www.mdpi.com/article/10.3390/jcm15103633/s1, Table S1: Search strategy in different databases; Table S2: List of excluded studies during the full-text screening phase and reasons for exclusion.

## Abbreviations

The following abbreviations are used in this manuscript:

BMS	Burning Mouth Syndrome

## References

[1] (2020). International Classification of Orofacial Pain, 1st edition (ICOP). Cephalalgia.

[2] Gobel H. (2018). The International Classification of Headache Disorders, 3rd Edition. https://ichd-3.org/.

[3] Jääskeläinen S.K., Woda A. (2017). Burning mouth syndrome. Cephalalgia.

[4] de Paiva J.P.G., Pedroso C.M., Muñoz R.M.L., Chmieliauskaite M., Villa A., Jorge J., Santos-Silva A.R. (2026). Prevalence of Associated Extraoral Symptoms and Comorbidities in Burning Mouth Syndrome Patients: A Systematic Review. Oral Dis..

[5] Marino M., Klasser G.D., Epstein J.B. (2020). Burning mouth syndrome and TMD overlap. J. Oral Facial Pain Headache.

[6] Musella G., Canfora F., Caponio V.C.A., Vardas E., Kouri M., Nikitakis N., Troiano G., Aria M., D’Aniello L., Lo Muzio L. (2025). Oral dysaesthetic and perceptual disorder, a distinct subset of chronic orofacial pain without burning symptoms: A case-control study. J. Oral Rehabil..

[7] (2018). Headache Classification Committee of the IHS. Diagnostic approach to BMS. Cephalalgia.

[8] Khan S.A., Keaser M.L., Seminowicz D.A., Nusbaum A.O., Gustin S.M. (2021). Neuroimaging findings in burning mouth syndrome: A systematic review. J. Dent. Res..

[9] Sun A., Wu K.M., Wang Y.P., Lin H.P., Chen H.M. (2013). Pharmacologic treatments for burning mouth syndrome: A systematic review. Oral Dis..

[10] Peters M.D.J., Godfrey C., McInerney P., Soares C.B., Khalil H., Parker D., Aromataris E., Munn Z. (2020). Methodology for JBI Scoping Reviews. JBI Manual for Evidence Synthesis.

[11] Tricco A.C., Lillie E., Zarin W., O’Brien K.K., Colquhoun H., Levac D., Moher D., Peters M.D., Horsley T., Weeks L. (2018). PRISMA Extension for Scoping Reviews (PRISMA-ScR): Checklist and Explanation. Ann. Intern. Med..

[12] De Siqueira S.R.D.T., Teixeira M.J., De Siqueira J.T.T. (2013). Orofacial pain and sensory characteristics of chronic patients compared with controls. Oral Surg. Oral Med. Oral Pathol. Oral Radiol..

[13] De Siqueira S.R.D.T., Manoel D.D.S., Teixeira M.J., De Siqueira J.T.T. (2014). Somatosensory investigation of patients with orofacial pain compared with controls. J. Neuropsychiatry Clin. Neurosci..

[14] Canfora F., Michelotti A., Barone S., Farella M. (2024). Occlusal tactile acuity in patients with burning mouth syndrome: A case-control study. Semin. Orthod..

[15] Sekine N., Okada-Ogawa A., Asano S., Takanezawa D., Nishihara C., Tanabe N., Imamura Y. (2020). Analgesic effect of gum chewing in patients with burning mouth syndrome. J. Oral Sci..

[16] Scala A., Checchi L., Montevecchi M., Marini I., Giamberardino M.A. (2003). Update on burning mouth syndrome: Overview and patient management. Crit. Rev. Oral Biol. Med..

[17] Toyofuku A. (2016). Psychosomatic problems in dentistry. Biopsychosoc. Med..

[18] Zakrzewska J.M. (2013). Multi-dimensionality of chronic pain of the oral cavity and face. J. Headache Pain.

[19] Grushka M., Epstein J.B., Gorsky M. (2003). Burning mouth syndrome and other oral sensory disorders: A unifying hypothesis. Pain Res. Manag..

[20] Corsalini M., Di Venere D., Pettini F., Lauritano D., Petruzzi M. (2013). Temporomandibular disorders in burning mouth syndrome patients: An observational study. Int. J. Med. Sci..

[21] Chimenos-Küstner E., de Luca-Monasterios F., Schemel-Suárez M., Rodríguez de Rivera-Campillo M.E., Pérez-Pérez A.M., López-López J. (2017). Burning mouth syndrome and associated factors: A case-control retrospective study. Síndrome de boca ardiente y factores asociados: Estudio retrospectivo de casos y controles. Med. Clin..

[22] Lamey P.J., Lamb A.B. (1988). Prospective study of aetiological factors in burning mouth syndrome. BMJ.

[23] Svensson P., Kaaber S. (1995). General health factors and denture function in patients with burning mouth syndrome and matched control subjects. J. Oral Rehabil..

[24] Dal Sacco D., Gibelli D., Gallo R. (2005). Contact allergy in the burning mouth syndrome: A retrospective study on 38 patients. Acta Derm. Venereol..

