# Supplementary material for: Exploring Masticatory and Occlusal Factors in Burning Mouth Syndrome: A Scoping Review
Source: J Clin Med. 2026 May 9;15(10):3633. doi: 10.3390/jcm15103633 (PMC13207299; doi:10.3390/jcm15103633)
Supplement: Supplementary file 1 [file jcm-15-03633-s001.zip › Supplementary Table S1.pdf]

Supplementary Table S1

Table S1. Search strategy in different databases.

Database	Date of last search	Full Search String (English)	Filters / Limits	Number of records retrieved
**PubMed (MEDLINE)**	February 28, 2026	(“chewing” OR “mastication” OR “dental occlusion” OR “tactile acuity” OR “dental dysesthesia”) AND (“burning mouth syndrome” OR “BMS”)	English language; no date restrictions	96
**Cochrane Library**	February 28, 2026	(“chewing” OR “mastication” OR “dental occlusion” OR “tactile acuity” OR “dental dysesthesia”) AND (“burning mouth syndrome” OR “BMS”)	English language; article and review records only	2
**Web of Science (Core Collection)**	February 28, 2026	(“chewing” OR “mastication” OR “dental occlusion” OR “tactile acuity” OR “dental dysesthesia”) AND (“burning mouth syndrome” OR “BMS”)	English language;	50
**Scopus**	February 28, 2026	(“chewing” OR “mastication” OR “dental occlusion” OR “tactile acuity” OR “dental dysesthesia”) AND (“burning mouth syndrome” OR “BMS”)	English language; document types: article, review;	86
