# Supplementary material for: Exploring Masticatory and Occlusal Factors in Burning Mouth Syndrome: A Scoping Review
Source: J Clin Med. 2026 May 9;15(10):3633. doi: 10.3390/jcm15103633 (PMC13207299; doi:10.3390/jcm15103633)
Supplement: Supplementary file 1 [file jcm-15-03633-s001.zip › Supplementary Table S2_rev2.pdf]

Supplementary Table S2

Table S2. List of excluded studies during the full-text screening phase and reasons for exclusion.

Reference	Reason for exclusion
Ciesielska A, Kusiak A, Ossowska A, Grzybowska ME. Changes in the Oral Cavity in Menopausal Women-A Narrative Review. Int J Environ Res Public Health. 2021;19(1):253. Published 2021 Dec 27. doi:10.3390/ijerph19010253	Participants do not present burning mouth syndrome
Hakeberg M, Berggren U, Hägglin C, Ahlqwist M. Burning mouth symptoms reported among middle-aged and elderly women. Community Dent Oral Epidemiol. 1997;25(5):350-354.	Study doesn't include evaluation of mastication
Chimenos Küstner E, Marques Soares MS. Burning mouth and saliva. Med Oral. 2002;7(3):173-178.	Study doesn't include evaluation of mastication
Virgili A, Corazza M, Trombelli L, Arcidiacono A. Burning mouth syndrome: the role of contact hypersensitivity. Oral Surg Oral Med Oral Pathol Oral Radiol Endod. 1996;82(3):306-310. doi:10.2340/0001555576488490	Study doesn't include evaluation of mastication
Skoglund A, Egelrud T. Hypersensitivity reactions to dental materials in patients with lichenoid oral mucosal lesions and in patients with burning mouth syndrome. Scand J Dent Res. 1991;99(4):320-328. doi: 10.1111/j.1600-0722.1991.tb01035.x	Study doesn't include evaluation of mastication
Domić I, Budmir J, Novak I, Mravak-Stipetić M, Lugović-Mihić L. Evaluation of food and food additive allergy in patients with angioedema, burning mouth syndrome, cheilitis, gingivostomatitis, oral lichenoid reactions, and perioral dermatitis. Acta Clin Croat. 2019;58(1):23-31. doi: 10.3390/cosmetics10010009	Study doesn't include evaluation of mastication
Steele JC, Bruce AJ, Davis MDP, Torgerson RR, Drage LA, Rogers RS. Clinically relevant patch test results in patients with burning mouth syndrome. Dermatitis. 2012;23(2):61-70. doi:10.1097/DER.0b013e31824a625e	Study doesn't include evaluation of mastication

Tinastepe N, Oral K. Neuropathic pain after dental treatment. Agri. 2013;25(1):1-6. DOI: 10.5505/agri.2013.55477	Participants do not present burning mouth syndrome
Scala A, Checchi L, Montevecchi M, Marini I, Giamberardino MA. Burning mouth syndrome: an overview. Minerva Stomatol. 2003;52(10):479-492. DOI: 10.1177/154411130301400405	Study doesn't include evaluation of mastication
Su N, Darling M, Grushka M. Evaluation of the mandibular function impairment questionnaire in clinical diagnosis of temporomandibular joint disorder. J Oral Rehabil. 2024;51(2):123-130.	Participants do not present burning mouth syndrome
Kagami H, Wang S, Hai B. Restoring the function of salivary glands. Oral Dis. 2008;14(1):15-24. doi: 10.1111/j.1601-0825.2006.01339.	Participants do not present burning mouth syndrome
Ibrahim N, Chamoun WT, El Outa A. Alexithymia, oral behaviors, and temporomandibular disorders: a dark triad? J Oral Rehabil. 2023;50(5):456-464.	Participants do not present burning mouth syndrome
Domić I, Budmir J, Novak I, Mravak-Stipetić M, Lugović-Mihić L. Assessment of allergies to food and additives in patients with angioedema, burning mouth syndrome, cheilitis, gingivostomatitis, oral lichenoid reactions, and perioral dermatitis. Acta Clin Croat. 2021;60(1):45-52. doi: 10.20471/acc.2021.60.02.14	Study doesn't include evaluation of mastication
Gao J, Chen L, Zhou J, Peng J. A case-control study on etiological factors involved in patients with burning mouth syndrome. J Oral Pathol Med. 2008;37(1):24-28. doi:10.1111/j.1600-0714.2008.00708.	Study doesn't include evaluation of mastication
Essick GK, Phillips C, Zuniga J. Effect of facial sensory re-training on sensory thresholds. J Oral Rehabil. 2007;34(6):408-415. doi: 10.1177/154405910708600616	Participants do not present burning mouth syndrome

Nalamliang N, Sumonsiri P, Thongudomporn U. Masticatory performance is influenced by masticatory muscle activity balance and the cumulative occlusal contact area. J Oral Rehabil. 2021;48(8):876-883. doi: 10.1016/j.archoralbio.2021.105113	Participants do not present burning mouth syndrome
Schimmel M, Voegeli G, Duvernay E, Leemann B, Müller F. Oral tactile sensitivity and masticatory performance are impaired in stroke patients. J Oral Rehabil. 2017;44(3):163-170. doi: 10.1111/joor.12482	Participants do not present burning mouth syndrome
Kiliaridis S, Tzakis MG, Carlsson GE. Short-term and long-term effects of chewing training on occlusal perception of thickness. Acta Odontol Scand. 1990;48(6):347-352. doi: 10.1111/j.1600-0722.1990.tb00955.	Participants do not present burning mouth syndrome
Scala A, Checchi L, Montevecchi M, Marini I, Giamberardino MA. Burning mouth syndrome: overview and patient management. Minerva Stomatol. 2003;52(10):479-492. doi: 10.1177/154411130301400405	Study doesn't include evaluation of mastication
López-Jornet P, Camacho-Alonso F, Andujar-Mateos P, Sánchez-Siles M, Gómez-García F. Salivary flow and pH in patients with burning mouth syndrome. J Oral Pathol Med. 2010;39(4):282-287. doi: 10.3748/wjg.v19.i5.665	Study doesn't include evaluation of mastication
Bergdahl M, Bergdahl J. Burning mouth syndrome: prevalence and associated factors. J Oral Pathol Med. 1999;28(8):350-354. doi:10.1111/j.1600-0714.1999.tb02052.	Study doesn't include evaluation of mastication
Ni Riordain R, Moloney E, O'Sullivan K, McCreary C. Quality of life in patients with burning mouth syndrome. J Oral Pathol Med. 2012;41(7):504-510.	Study doesn't include evaluation of mastication
Eliav E, Heir GD, Wenacur R, Shanti SJ, Dorfman Z, Benoliel E, Gracely R, Benoliel R. Evidence for neuropathic pain mechanisms in burning mouth syndrome. J Orofac Pain. 2007;21(4):336-346.	Study doesn't include evaluation of mastication
Werfalli S, Drangsholt M, Johnsen JM, Jeffrey SK, Dakhil S, Presland RB, LeResche L. Saliva flow rates and clinical characteristics of patients with burning mouth syndrome: a case-control study. *Int J Oral Maxillofac Surg*. 2021;50(9):1234-1240. doi:10.1016/j.ijom.2021.01.018	Study doesn't include evaluation of mastication
